# Biomedical applications of graphene-based nanomaterials: recent progress, challenges, and prospects in highly sensitive biosensors

**DOI:** 10.1186/s11671-024-04032-6

**Published:** 2024-06-17

**Authors:** Arabinda Baruah, Rachita Newar, Saikat Das, Nitul Kalita, Masood Nath, Priya Ghosh, Sampath Chinnam, Hemen Sarma, Mahesh Narayan

**Affiliations:** 1 Department of Chemistry, Gauhati University, Guwahati, Assam 781014 India; 2 Indian Institute of Technology Guwahati, Guwahati, Assam 781039 India; 3 University of Technology and Applied Sciences, Muscat, Oman; 4 Department of Chemistry, M.S. Ramaiah Institute of Technology (Autonomous Institution, Affiliated to Visvesvaraya Technological University, Belgaum), Bengaluru, Karnataka 560054 India; 5 Department of Botany, Bodoland University, Rangalikhata, Deborgaon, Kokrajhar (BTR), Assam 783370 India; 6 Department of Chemistry and Biochemistry, University of Texas at El Paso, UTEP, 500 W. University Ave, El Paso, TX 79968 USA

## Abstract

Graphene-based nanomaterials (graphene, graphene oxide, reduced graphene oxide, graphene quantum dots, graphene-based nanocomposites, etc.) are emerging as an extremely important class of nanomaterials primarily because of their unique and advantageous physical, chemical, biological, and optoelectronic aspects. These features have resulted in uses across diverse areas of scientific research. Among all other applications, they are found to be particularly useful in designing highly sensitive biosensors. Numerous studies have established their efficacy in sensing pathogens and other biomolecules allowing for the rapid diagnosis of various diseases. Considering the growing importance and popularity of graphene-based materials for biosensing applications, this review aims to provide the readers with a summary of the recent progress in the concerned domain and highlights the challenges associated with the synthesis and application of these multifunctional materials.

## Introduction

In the past few decades, research on functional nanomaterials has become exceedingly popular due to their easily tunable physicochemical and optoelectronic properties [1–7]. Among diverse types of nanostructured materials, predominantly two-dimensional materials have gained exceptional research thrust because of their tremendous application potential in different spheres of science. In the scientific literature, numerous reports can be found investigating the properties and applications of two-dimensional nanomaterials [8, 9]. Graphene (GR) is an allotrope of carbon consisting of *sp*^*2*^ hybridized carbon atoms with a monolayer of thickness ranging between 0.34 to 0.5 nm and an average carbon–carbon bond length of 1.42 Å [10]. Notably, the number of reports on graphene-related research started rising exponentially only after 2010, when Andre Geim and Konstantin Novoselov were jointly awarded the Nobel Prize in physics for their ground-breaking work.

Graphene and related materials, such as graphene oxide (GO), reduced graphene oxide (rGO), graphene-based nanocomposites, and graphene quantum dots (GQD), owing to their unique and advantageous physical and chemical properties, are being increasingly used in applications like wastewater treatment, photocatalysis, disinfection, carbon dioxide sequestration, battery applications, supercapacitors, electrochemical sensors, nano biosensors, seawater desalination, food processing, package engineering, natural gas separation, water oxidation, hydrogen generation and storage, membrane filtration, and pollutant adsorption [11, 12].

Graphene, a two-dimensional carbon nanostructure, possesses exceptional properties like high mechanical strength, electrical conductivity, and thermal stability due to its hexagonal lattice structure. These qualities make it highly reactive and versatile for various applications. The ease of preparation of GO from graphite powder and scopes for surface modification makes it a fascinating material for various applications [12]. Nanocomposites based on graphene or graphene oxide are also easy to prepare. Its large surface area enables efficient chemical functionalization and the formation of strong bonds with other materials, while its outstanding electrical and thermal conductivity enhances compatibility for creating advanced nanocomposites with tailored properties. The graphene oxide surface is rich in oxygen-containing functionalities, such as hydroxyl, epoxy, carboxyl, and carbonyl. So, it can rapidly bind with various materials like metals, metal oxides, polymers, ceramics, and biomolecules. This facilitates the facile production of different graphene-based nanocomposites with a broad range of applications.

Biosensors are sensing platforms that utilize an enzyme, antibody, aptamer, or other biomolecule to detect the target analyte [13]. Biorecognition molecules can be called nano biosensors if they are immobilized on nanomaterials. These nanomaterials act as signal transducers, and their high surface area helps enhance the nanobiosensors’ sensitivity. Various nano biosensors have been reported utilizing various sensory components and transducer materials in the literature. The most commonly used biorecognition entities are enzymes, antibodies, nucleic acids, aptamers, and supramolecular complexes. The sensory layer is designed based on the specificity of the bioreceptor towards the targeted analyte. The transducer layer in nano biosensors may consist of quantum dots, nanotubes, nanowires, nano-sheets, dendrimers, nanocomposites, or any nanostructured material.

Graphene and its derivatives are widely employed to engineer innumerable highly efficient biosensing platforms owing to their favourable mechanical and electronic properties. Such nano biosensors have proven highly significant in biomedical research, mainly in monitoring human health, analyzing food, and sensing environmental pollutants [13]. Graphene (GR) and its derivatives are extensively utilized as the transducer element in designing different nano biosensors. Moreover, based on the type of signal generated after biorecognition, nano biosensors are further arranged into different categories like optical, mechanical, electrical, electrochemical, and calorimetric nano biosensors. Integration of these nano biosensors into electronic devices, like smartphones, unveils newer possibilities of point-of-care testing and diagnosis [14].

In the present chapter, we have methodically summarized some of the most intriguing reports on nano biosensors, where graphene or its derivatives have been used as the signal transducer material. Graphene-based biosensors are most frequently used as optical or electrochemical sensors for detecting various pathogenic bacteria, viruses, and other biomarkers produced in the body [15]. Besides electrical, optical, and electrochemical sensors, owing to its high electrical and thermal conductivity and high elasticity, this wondrous material also allows the fabrication of mechanical and thermal sensors [15].

Here, for the benefit of readers from non-material science backgrounds, we have presented a concise discussion in the initial part of the chapter dedicated to the synthesis and attributes of GR-based materials. In the following sections, the utilization of graphene-based nanosensors for detecting heavy metals, various pathogens, and cancer biomarkers has been discussed in detail. In the last section of the chapter, preparation, application, and properties of the nanosensors developed by immobilizing different types of receptors, like enzymes, aptamers, peptides, and ani-bodies on graphene or its derivatives, have been elucidated with the help of most relevant and recent examples.

## Types and synthesis of graphene-based nanostructures (GBNs)

**Fig. 1:**
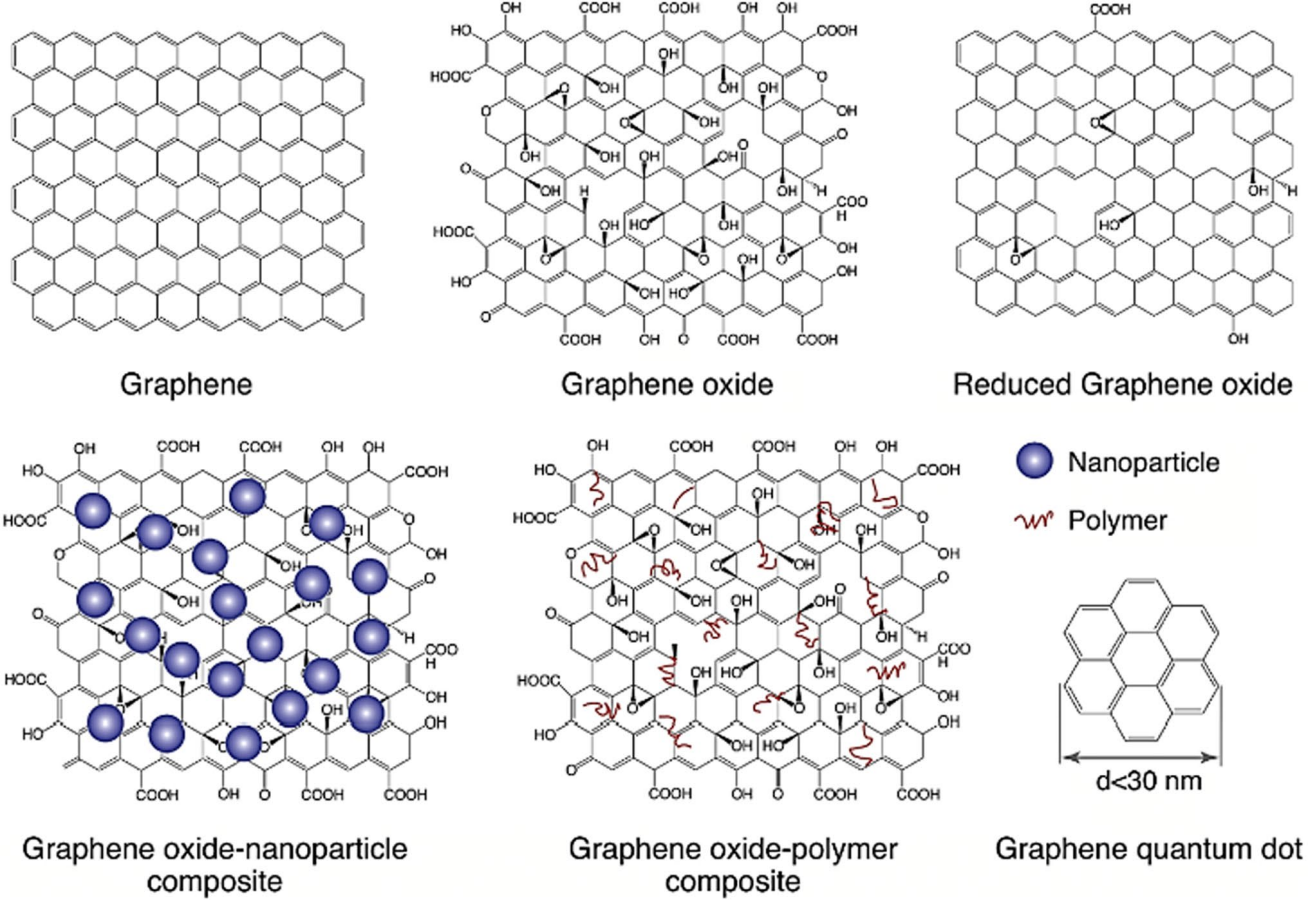
Different GR-based nanomaterials are illustrated schematically and reprinted with permission [16] Copyright Elsevier 2017

Graphene-based nanomaterials (GBNs) encompass a broad range of composites comprising GR and its derivatives. The starting materials for GBNs can either be graphene or graphite, from which various graphene derivatives are derived, such as GO, rGO and GQDs (Fig. 1). These derivatives possess distinct properties discernible by size, number of layers, and surface characteristics, rendering them unique and suitable for diverse applications in nanotechnology, materials science, and other scientific disciplines. The versatile nature of GBNs allows for tailoring their properties according to specific requirements, making them promising candidates for various advanced technological implementations.

### Graphene oxide (GO)

GO, an oxidized form of graphene obtained by oxidizing graphite is identified by the existence of epoxide, hydroxyl, and carboxylic groups on its graphene sheets [17]. The synthesis of GO is commonly achieved through Hummer’s method or its variations, involving the oxidation of graphite to varying degrees using sulfuric acid and potassium permanganate. The resulting GO primarily consist of hydroxyl and epoxy groups on the basal plane, while carbonyl, carboxyl, lactone, and phenol groups are found in lower quantities at the sheet edges [18–20]. The carboxylic acid groups contribute to a negatively charged surface, imparting stability in polar liquid mediums, while the epoxy and hydroxyl groups establish H-bonding and weak associations with other functional groups. Consequently, GO exhibits excellent miscibility with water and other polar solvents, making it highly versatile and promising for numerous applications in nanotechnology, biomedicine, and materials science [21].

### Reduced graphene oxide (rGO)

The transformation of GO into rGO involves the elimination of oxygen moieties, leading to the restoration of the conjugated structure in the GR sheets [22]. A variety of reducing agents, like hydrazine [23], sodium borohydride [24], ascorbic acid [25], and hydroquinone [26], are employed in the chemical treatment of GO to achieve the reduction process. Consequently, rGO exhibits enhanced electrical conductivity compared to GO, attributed to reestablishing a well-connected graphitic network with fewer disruptions [27]. Despite these changes, rGO retains its biocompatibility, making it a favourable candidate for biomedical applications, including tissue engineering, biosensing, and cell culture [28, 29].Moreover, rGO’s unique physicochemical properties enable noncovalent interactions, like Van Der Waals interactions and π–π stacking, allowing for the physical adsorption of both polymers [30] and small molecules [31] onto its surface. These versatile characteristics of rGO open up new avenues for advancing various biomedical and materials science research endeavours [28, 29].

### Graphene quantum dots (GQDs) and nano-rGOs

GQDs and nano-rGOs comprise one or multiple layers of GR, with lateral sizes typically below 30 nm, ranging from a monolayer to around ten layers [32, 33].GQDs can be produced through bottom-up and top-down processes. Top-down methods associated with segmenting large GR sheets into ultra-small GQDs through various electrochemical, chemical, or physical processes include electron beam lithography [34], hydrothermal treatment [35], electrochemical oxidation [36], and chemical exfoliation [37]. Conversely, bottom-up strategies focus on synthesizing GR sheets with a defined number of conjugated C-atoms from aromatic molecules. Techniques like cage opening of fullerene [38], pyrolysis [32], oxidative condensation processes [39], and pyrene nitration [40] are commonly employed in bottom-up GQD synthesis.

The distinctive properties of GQDs stem from edge effects and quantum solid confinement effects, resulting in powerful intrinsic photoluminescence. These characteristics may be modulated by manipulating the size, defects, functionalities and shape of GQDs [41, 42]. Moreover, GQDs offer unique benefits compared to traditional semiconductor quantum dots, such as reduced toxicity and enhanced biocompatibility, attributed to the absence of heavy metals commonly employed in traditional quantum dot synthesis [43, 44]. Consequently, GQDs find extensive application in photodynamic therapy, drug delivery, cellular imaging, and biosensing [45–48]. These unique properties and biocompatibility position GQDs as tantalizing candidates for advancing optoelectronic and biomedical technologies. The unique combination of size-dependent luminescence and facile surface functionalization renders GQDs and nano-rGO promising candidates for advancing cutting-edge technologies in various scientific and engineering disciplines.

## Graphene-based composites

In its pure form, graphene is inherently hydrophobic and exhibits limited solubility in most solutions. However, in the production of graphene composites, achieving solubilization becomes a crucial aspect. To enrich the solubility of GR, numerous functionalization tactics have been employed, involving chemical [49, 50], covalent [51, 52], or noncovalent [53, 54] attachment of different functional groups to the carbon backbone of graphene. These modifications introduce polar or hydrophilic moieties, rendering the graphene more amenable to dispersion and interaction with solvents or matrices. By tailoring the functionalization approach, researchers can effectively overcome the inherent hydrophobic nature of graphene, unlocking its potential for broader applications in nanotechnology, and biomedicine.

### Graphene–polymer composites

GR and its derivatives play a pivotal role as filler materials in polymer composites because of their spectacular mechanical strength, exceptional thermal stability, and superior electrical conductivity. The overall performance and characteristics of GR-polymer composites are influenced by the quality of the polymer matrix and graphene filler and factors such as filler dispersion, filler-matrix bonding, and the filler-to-matrix ratio. These aspects are heavily influenced by the production methods employed. In-situ polymerization [55], melt blending [56], and solution mixing [57] are the fundamental techniques utilized for the creation of GR-polymer composites, mirroring the processing methods employed for conventional polymers. Achieving an optimal dispersion of graphene fillers in the polymeric structure and establishing a solid interface between the two components is crucial to maximizing the entire potential of GR-based composites, paving the way for utilization in various sectors like aerospace, structural engineering and electronics. GBNs can also serve as nanostructuring agents and influence cellular mechanosensing. They enable the creation of effective bio interfaces, fostering the development of nanosensors for detecting bacterial cells and modulating biological effects in biomedical composite applications [11]. As a result, GBNs are increasingly utilized in biomaterials engineering, offering versatile solutions across biomedical and biotechnological domains, with potential implications for healthcare and beyond.

### Graphene-metal nanoparticle (NP) composites

GR-metal nanoparticle (NP) composites find extensive utility in diverse disciplines, like electrochemical sensing [58], SERS (surface-enhanced Raman scattering) [59] and catalysis [60] depending on the type of anchoring nanoparticles utilized. Various synthesis methods are employed to create these composites, including thermal evaporation [61], photochemical synthesis [62], microwave-aided synthesis [63], chemical reduction [64], and electroless metallization [65].

Among these methods, straightforward chemical reduction of metallic substances accompanied by GO or rGO suspensions stands out as a straightforward approach for fabricating graphene-metal NP composites. The defects and residual oxygenated moieties in rGO and GO serve as nucleation zones for forming metal nanoparticles, facilitating the incorporation of metal nanoparticles into the GR matrix. For instance, a GR-Au NP composite was successfully produced through NaBH_4_-mediated reduction of AuCl_4_ in a rGO-ODA (octadecyl amine) solution [66].

### Graphene-semiconductor nanomaterial composites

Graphene-semiconductor nanomaterial composites have attracted considerable interest owing to their vast utility in applications related to energy, optics, and electronics devices, including Li-ion batteries, supercapacitors, and solar cells. A diverse range of semiconductor nanomaterials, including TiO_2_ [67], ZnO [68], SnO_2_ [69], MnO_2_ [70], Co_3_O_4_ [71], Fe_3_O_4_ [72], NiO [73], Cu_2_O [74], RuO_2_ [75], CdS [76], and CdSe [77], have been successfully developed and integrated onto GR-based templates. These composite materials are fabricated through various synthetic methods, such as microwave-assisted growth [78], solution mixing [71], vapour deposition [73], in-situ crystallization [79], and electrochemical deposition [80]. The in-situ crystallization procedure has emerged as a prominent method for fabricating composites consisting of GO or rGO with semiconductor nanoparticles. This technique combines GO or rGO with the corresponding semiconductor precursor, such as Cd(CH_3_COO)_2_, in a suitable solvent like dimethyl sulfoxide (DMSO). The mixture is then subjected to hydrothermal treatment inside an autoclave at a specific temperature and duration, typically 180 °C for 12 h. During this process, two essential transformations occur concurrently: the hydrothermal reaction leads to the formation of semiconductor nanoparticles (NPs) of CdS, and simultaneously, GO is reduced to rGO within the DMSO medium, which functions both as a solvent and a sulfur source [79]. This in-situ synthesis strategy enables the creation of graphene-semiconductor composites, where the semiconductor NPs are intimately anchored on the graphene surface, fostering synergistic effects, and enhancing the composite’s performance for a spectrum of possible uses including energy conversion devices catalysis, and optoelectronics.

## Nanotechnology mediated biosensors

Nanotechnology and nanomaterials are revolutionizing biosensor development with scalable nanofabrication processes, enabling high-yield biosensors with varied applications in healthcare, pharmaceuticals, diagnostics, drug delivery, and environmental monitoring. Nanobiosensors, especially those using optical fibres and terahertz-domain metasurfaces, have significantly advanced in refractometric analysis, biomolecular detection (labelled and label-free), and medical technology.

Rahman et al*.*[81] have made noteworthy contributions, inspiring researchers to embrace nanotechnology-mediated biosensors for biomolecular analysis and medical progress. Biosensor technology based on nanostructures, particularly graphene-based biosensors, is a potent and rapidly advancing development area. These biosensors offer early detection of malignancies, improving patients’ access to early diagnostic and treatment options and enhancing their quality of life. GR-based biosensors, like electrochemical biosensors, FET (field-effect transistors), SPR (surface plasmon resonance) and fluorescence sensors demonstrate superior sensitivity, stability, selectivity, and wide detection ranges. Additionally, derivatives of GR, like GQDs, rGO, and GO have shown promising properties and applications in detecting cancer early through nano biosensors. However, specific challenges must be addressed during development and implementation [82]. Nanobiosensing has undergone a revolution caused by fluorescent nanosensors. Fluorescent-based bio probes have been utilized to develop highly responsive nano biosensors to identify various chemical and biological agents. This is because of their high surface-to-volume ratio, selectivity, superior sensitivity, and variable electronic and optical features. In this initial study, Saman et al*.*[83] reviewed all of the most recent advancements regarding the application of fluorescent-based nanoprobes to examine a variety of biological substances and microorganisms. Furthermore, they discussed insight into the structural design and mechanistic methods employed to create nanosensors that could identify things like cancer biomarkers, antioxidants, enzymes, amino acids, heavy metals, proteins, carbohydrates, urine metabolites, blood gases, toxins, medicines, etc.

The recent SARS-CoV-2 epidemic has once again demonstrated that viral diseases endanger life on Earth. Virus-related illnesses raise public health worries. The traditional molecular approaches are currently the norm for virus detection, but they require expensive machinery and specialized workers. The development of nano biosensors have shown significant potential in the field of sensing. Colourimetric biosensors, photonic crystal biosensors, fluorometric biosensors, electrochemical biosensors, SPR biosensors, and optical biosensors are just a few detection methods developed using biosensors. This review makes use of recently developed biosensors to treat viral infections. Nanobiosensors have a promising future in biological research since they can detect the analytes more precisely, quickly, and affordably [84]. For detecting cancer, observing tumour progression, and tracking tumour evolution, liquid biopsies are a highly viable substitute for traditional tissue biopsies. In recent years, extracellular vehicles (EVs) generated by tumours have emerged as yet another liquid specimen’s diagnostics source. It is now evident that EVs facilitate intercellular communication and play a crucial part in several healthy and pathological processes, including cancer, even though it was once believed to be a cellular waste.

Gracia et al.[85] addressed the current state of the science for nanomaterial-based biosensors and talked about recent advancements in the recognition of EVs in this review. The dominant biosensors based on nanotechnology use nanomaterials as a recognition aspect to increase their sensitivity. However, nanotechnology-based biosensors are another name for the biosensors created by applying nanofabrication techniques. Nanotechnology refers to using nanoscience to create beneficial goods, whereas nanoscience focuses on methodologies and procedures for creating and characterizing nanoscale materials, constructions, and devices. Yazdi et al*.*[86] concentrated on creating various nanoparticles with various shapes and building materials.

### Importance of graphene-based nano biosensors in biomedical research and recent progress

One of the most recent global trends is the creation of innovative biosensors. Introducing biosensors has increased interest in monitoring infectious disorders, particularly those caused by microbial pathogens, including fungi, bacteria, and viruses. Hassan et al.  [87] used these examples to highlight the fundamentals of nanomaterials and their effects on the design and construction of nanosensors and biosensors. As an effective analytical instrument, biosensors have been applied in essential fields like biomedical diagnostics, disease monitoring, and environmental analysis. Nanotechnology has advanced dramatically in the biological and pharmaceutical fields and can now cure and diagnose various illnesses while overcoming numerous challenges. The biomedical and pharmaceutical industries have seen exponential growth in nanotechnology, which can potentially solve many problems in disease prevention, treatment, and diagnosis. Yadav et al*.*[88] presented insight into various diagnostic techniques centred on nanoparticles that might have been developed for the speedy and early identification of such diseases. Nanomaterial-based biosensors have enabled the development of improved biosensor capabilities, like high sensitivity, selectivity, and low limits of detection (LODs). Silicon and gold nanoparticles have been successfully developed to improve DNA sensors for MERS and COVID-19. According to Rabbania et al*.* nanotechnology is one of the booming industries of the current time, and nanotechnology is becoming more and more pervasive in most spheres of life. Numerous industries, including medicine, optics, energy research, and magnetism, are thriving due to advancements in nanotechnology. Currently, various nanomaterials are utilized in medicine delivery methods, antimicrobial drugs, biolabeling, and sensors. Nanobiosensors have numerous applications due to their extreme adaptability and versatility. Detecting serum antigens, carcinogens, and several metabolic diseases uses nano biosensors. Serum analysis is necessary to diagnose numerous conditions, including diabetes, cancer, and allergic reactions [89]].

Numerous nanomaterials are currently Nanobiosensors primarily used in approximating and diagnosing health-related in-vivo properties. Developing biosensors based on nanomaterials has become a significant area in the diagnostics industry. Four nanomaterials, including gold nanoparticles, graphene, carbon nanotubes, and photonic crystals, have gained popularity in biosensor research due to the rising demand for devices with increased sensitivity and selectivity, quick response times, and inexpensive costs. Su et al*.* outlined the most current technological advancements utilizing the previously mentioned nanomaterials for the electrochemical sensing of biomolecules like C_6_H_12_O_6_, DNA, protein, toxins, and so on [90].

### Applications in disease diagnosis and biomolecule sensing-major types of biosensors

There are several different categories that nano biosensors could fit into. Due to this nature, nanoparticles are used in the biosensing process. They are classified as optical biosensors, electrical biosensors, and electrochemical biosensors.

#### Optical biosensors

The detection and measurement of analytes using optical biosensors depends on measuring light interactions. These biosensors may track the binding processes between the biological component and the target analyte using concepts like absorbance, fluorescence, or surface plasmon resonance (SPR).

Optical biosensors have an excellent history of identifying biological systems, and they have significantly advanced environmental monitoring, food process management, drug development and clinical diagnostics. Chen and coworkers stated that the recent developments in optical biosensors with a particular emphasis on configurations based on (1) SPR-based biosensors, (2) optical waveguides, (3) optical resonators, (4) photonic crystals, and (5) optical fibre. Both sensor design and bio-specific coatings in optical biosensor technologies have advanced substantially. Several chemical and biological analytes have been detected using optical biosensors [91]. Ji et al. innovatively developed optical biosensors and various applications by leveraging GR and its derivatives as sensing components for identifying a variety of biomolecules, including antigen antibodies, proteins, nucleic acids, cellular secretions, and single cells. These new exceptionally efficient optical sensors have the potential to notice shifts in biomolecular interactions and surface structure with the benefits of rapid detection and high sensitivity [8]. Optical biosensors offer a strong probability for identifying several kinds of cancer since they are rapid, real-time, portable, sensitive, and have a modest diagnostic limit. Kaur et al*.*[92] concentrate on new developments in biosensors with optical properties for earlier cancer identification. The graphene oxide-aptamer complex (GO-apt) has been developed by Tan and his associates for the detection of CCRF-CEM (CCRF-CEM cells are human T lymphoblasts). According to the variations in fluorescence signals, they managed to quantify CCRF-CEM cells across a broad spectrum, including 1 × 10^2^–1 × 10^7^ cells/mL along with a notable limit of detection (LOD) of 10 cells/mL [93]. Cheng et al. presented a FRET-based biosensor utilizing DOX (doxorubicin)-enhanced GO to detect Dopamine in various mediums like cells, aqueous solutions and human serum. This DOX-GO fluorescent sensor exhibited significant accuracy in detecting dopamine, with a concentration range between 8.3 × 10^–7^ and 3.3 × 10^–5^ M in aqueous solutions and 1.44–11.48 μmol L^−1^ in human serum. Notably, a linear trend was identified when comparing the fluorescence intensity ratio (F_o_/F) of DOX before and after the introduction of GO, covering GO concentrations from 0.5 to 20 μg mL^−1^ at 598 nm [94].

**Fig. 2:**
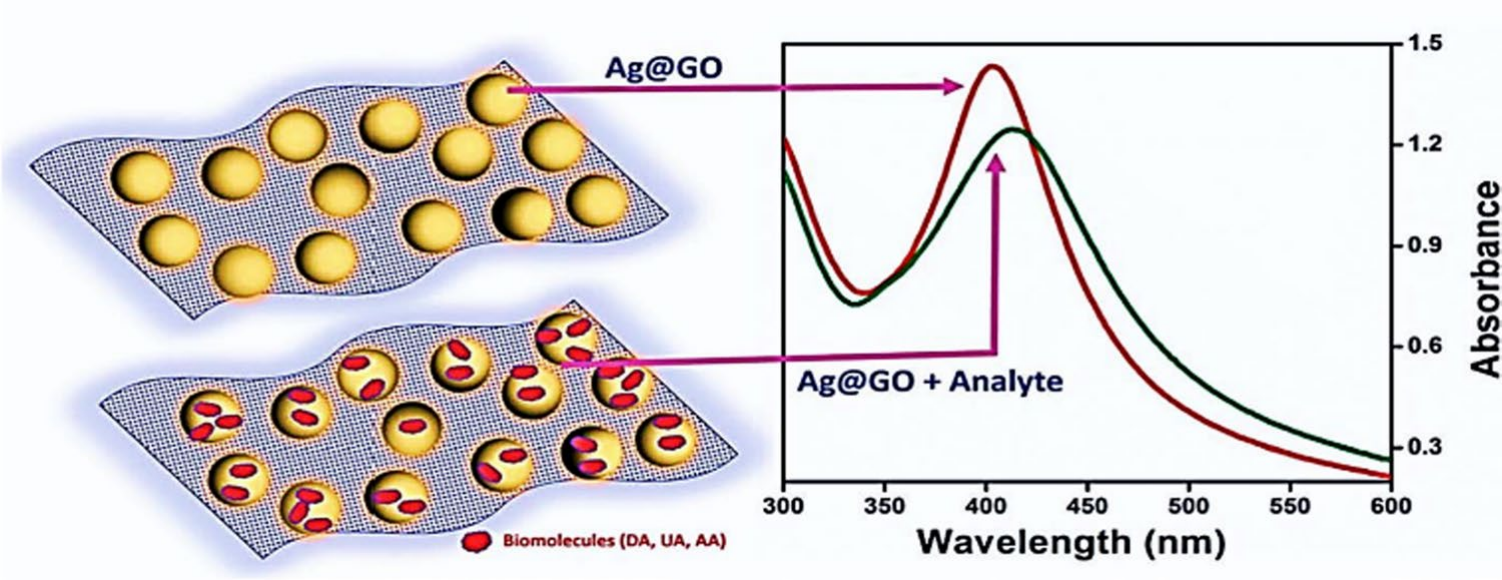
Pictorial representation of biomolecular optical detection using the Ag@GO nanocomposite. [95] Adapted from the article

Modern biomedical applications require optical sensing technology because it offers quick and practical techniques to identify desired analytes across various samples. Further advantages of optical-based biosensors are their high sensitivity, dependability, resilience, and potential for integration onto a single chip [96]. Kamali et al*.* have developed silver nanoparticles anchored graphene oxide nanocomposite via a simple chemical reduction technique and employed it as an optical biosensor for detecting biomolecules like ascorbic acid (AA), uric acid (UA) and dopamine (DA) as depicted in Fig. 2. [95] Yin et al. has proposed a graphene-dielectric metasurface based efficient optical biosensor. This metasurface consists of an assortment of asymmetric silicon bars laid down onto a graphene layer. These bars create a Q-factor (high-quality factor) Fano resonance, characterized by a sharp optical response. Each silicon bar includes a gap that confines the optical field, enhancing interaction with analytes. The metasurface's geometric properties are optimized for performance. The graphene layer serves as a transducer with strong biomolecule adsorption and immobilization capabilities. Operating at 1.55 μm wavelength, the metasurface's Fano resonance achieves a sensitivity of 392 nm/RIU due to tightly confined optical fields between the gaps of the asymmetric silicon bars. High figures of merit (FOM) and linearity (R^2^) values, estimated at 587 RIU-1 and 0.999 respectively, indicate excellent sensor performance. This proposed structure holds promise as a valuable biosensor for detecting crucial biomolecules in biomedical applications, including glucose, ssDNA, and haemoglobin [97].

Panda et al*.* conducted a detailed analytical study on an N-FK51A glass prism-based surface plasmon resonance sensor coated with graphene. This sensor operates at 589 nm wavelength and demonstrates effective detection of glucose in human blood serum (25–175 mg/dl) and gas analytes with variable refractive index (1.0000–1.0007). The sensor's performance is optimized by adjusting the Au layer thickness and GR layers. Sharp SPR reflectance curves are achieved through transfer matrix calculations and angular interrogation methods. The interface between graphene and the sensor layer enhances the electric field, leading to increased glucose and gas analyte absorption. Performance metrics including quality factor, detection accuracy, sensitivity, and resonance angle shift are evaluated with traditional SPR sensors. Replication results reveal notable outcomes: quality factor of 230.2, the detection accuracy of 2.55/°, the quality factor of 76.2 for glucose, detection accuracy of 1.41/° and sensitivity of 92.1°/RIU, and sensitivity of 275.15°/RIU for gas analytes. The presence of graphene yields high sensitivity due to significant field enhancement, with monolayer graphene exhibiting the highest sensitivity among various graphene layer thicknesses [98].

Goswami et al*.* present an economical and user-friendly approach for the rapid detection of low-level l-dopa in biological samples. The method relies on aggregation-induced emission amplification using reduced graphene oxide quantum dots coated with silk fibroin. The fluorescence intensity gradually increases upon l-dopa presence. The LOD of 76.18 nM within a linear spectrum of 0–35 M was established by evaluating the sensor’s turn-on efficiency at varying l-dopa concentrations. A smartphone-based device incorporating a 365 nm LED beneath the camera captures the changing fluorescent colour of the solution. Analyzing the red, green, and blue values through a mobile app revealed a matching LOD of 0.29 M within a similar concentration range. This economical and swift screening technique, suitable for remote areas lacking sophisticated instrumentation, holds promise for on-the-spot analyte detection [99].

#### Electrical biosensors

Electrical biosensors are research tools that use electrical sensing and biological principles to identify and examine biological substances or events. These sensors are made to transform an electrical signal which can be calculated, quantified, and analyzed from a biological signal or interaction. Applications for electrical biosensors include environmental monitoring, food safety, medical diagnostics, and biological research. Features like high sensitivity, quick reaction, mobility, and miniaturization are promising tools in various research and technology disciplines.

Neurotransmitters serve as chemical transmitters that control physiological and psychological processes in humans. Since the physiologically and therapeutically necessary content of neurotransmitters is frequently low (nM), electronic and electrochemical sensors for neurotransmitter detection play a significant part in securing selective and sensitive detection. The sensors also benefit from being wireless, small, and multichannel, opening up tremendous possibilities for implanted, long-term sensing capabilities that are impossible with spectroscopic or chromatographic detection methods. He et al*.*[100] covered and summarized recent developments in multiplex sensing, stretchy electrodes, microelectrode arrays, nanopipettes, and nanowires as sensors. Park et al*.*[101] established a COVID-19 screening biosensor built around an incredibly sensitive and portable virus receptor. To overcome the limited sensitivity of virus receptor-based biosensors, a dual gate field-effect transistor was selected. A synthetic virus was used to develop and optimize the FET biosensor. They are creating artificial viruses with characteristics similar to SARS-CoV-2, including a bilayer structure and size. Utilizing proteins and artificial viruses, the performance of the constructed biosensor was confirmed. Lim et al*.*[102] explored the potential of employing CuZn as a drug carrier for an electrical biosensor in drug delivery systems. The major component of semiconductors, CuZn, shows significant potential as an electrochemical detector to initiate API (active pharmaceutical ingredient). A green metal framework, which functions as an anionic node in polymers which are conductive, connected by bioactive ligands, is used to create this CuZn biosensor. The electrochemical performance of Cu, Zn, and their oxides as electrical biosensors to electrically trigger API is a crucial aspect of these metals' investigations.

Electrically powered biosensors are being pushed towards quick and effective detection that boosts the device’s functionality. Continuing research in nano- and material sciences has resulted in the inflexion of nanomaterial qualities that fit the trend parallel to the evolution of biosensors. Nadzirah et al.[103] stated that the quantity of scientific studies into biosensors with better analytical qualities and features has expanded because of TiO_2_ nano-hybrids. Due to the reflection of distinctive qualities for exceptional detection, incorporating TiO_2_ nano-hybrid in biosensors has received significant consideration in creating an ideal biosensor. Based on the most recent research, the various TiO_2_ nano hybrid biosensors' functions were demonstrated.

Avaz et al*.* constructed a graphene-based nanosensor and designed it for the targeted detection of NTO (nitrotriazolone) molecules using a molecularly imprinted film through basic electrical assays. Molecular imprinting forms distinct binding sites on the surface of polymeric films tailored to particular template molecules.

**Fig. 3:**
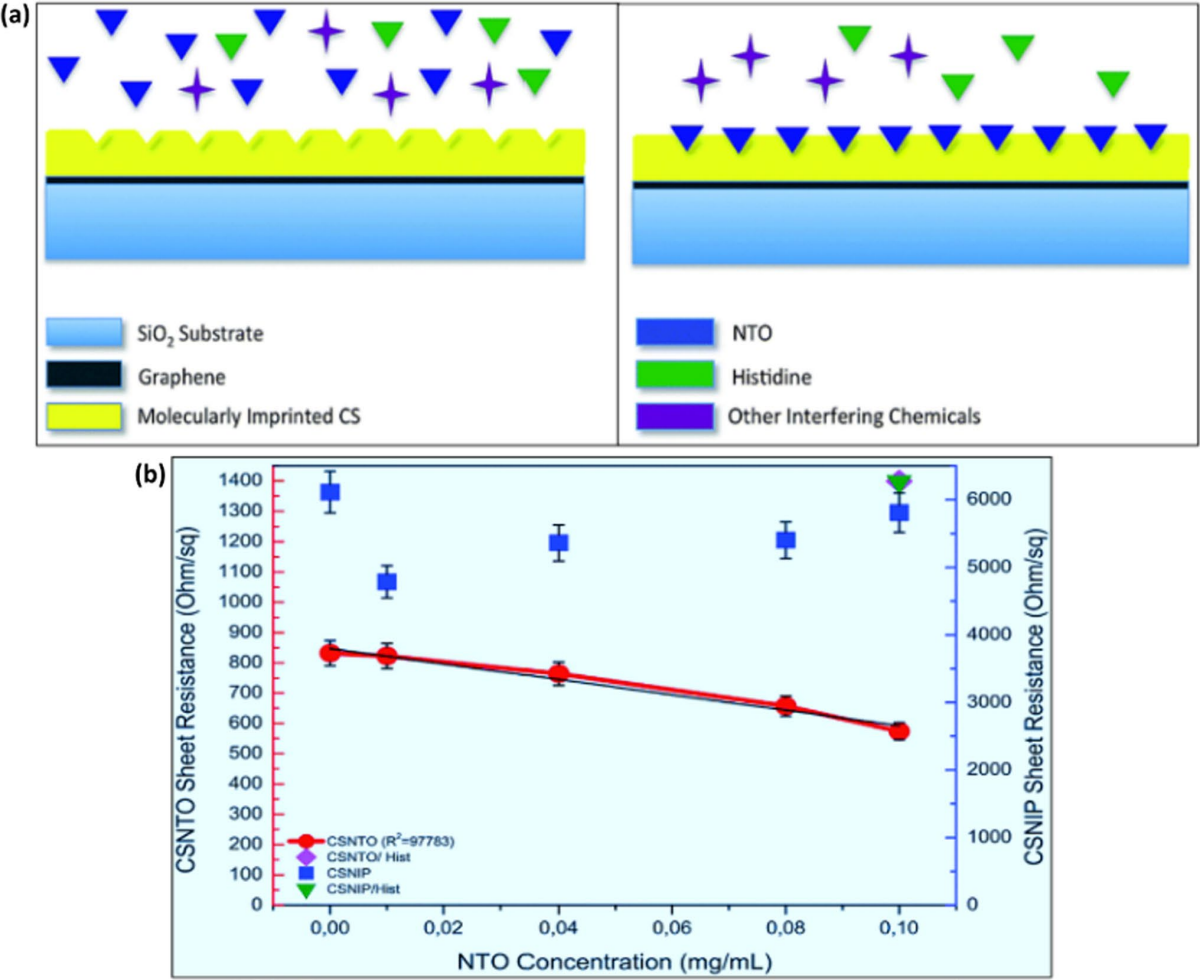
**a** Illustrative depiction of the molecular imprinting and recognition mechanism utilized within the nanosensor, **b** Comparative study for the selectivity of non-imprinted (CSNIP) sensor and imprinted (CSNTO) sensor for NTO and histidine (The blue scale applies to histidine values as well). [14, 104] Adapted from an open-access article

In the conventional method, these template molecules are incorporated during the polymer's crosslinking phase. Concurrently, the crosslinked polymer accumulated around the template, creating the particular binding positions. These binding positions engaged with the analyte using both a lock-and-key mechanism and through additional secondary interactions. This leads to the formation of highly specific polymer films or matrices. Upon encountering the analyte, the polymer film can selectively and reversibly absorb these molecules. Such absorption amplifies the charge within the film, subsequently affecting the resistance of the underlying graphene layer. A detailed depiction of this sensing approach via molecular imprinting is provided in Fig. 3a. The distinction between the sheet resistance measurements from the sensor embedded with NTO and the non- imbedded sensor is depicted in Fig. 3b. It was noted that the sheet resistance levels from the non-imbedded polymer were generally greater than those from the imbedded variants. This could be due to the embedded polymer's tendency to adsorb various species from the solution without selectivity [104].

#### Electrochemical biosensors

An electrochemical biosensor is a specialized biosensor designed to detect biological substances by converting their data into electrical signals like voltage, current, or impedance. One notable example is Clark’s pioneering work in developing the first electrochemical biosensor to determine glucose (C_6_H_12_O_6_) levels in human blood samples. This technology has broad applications in detecting various biological entities like enzymes, viruses, proteins and antibodies. Electrochemical biosensors are seeing increasing interest and development in various domains, including biology, electronics, material science, and engineering.

**Fig. 4:**
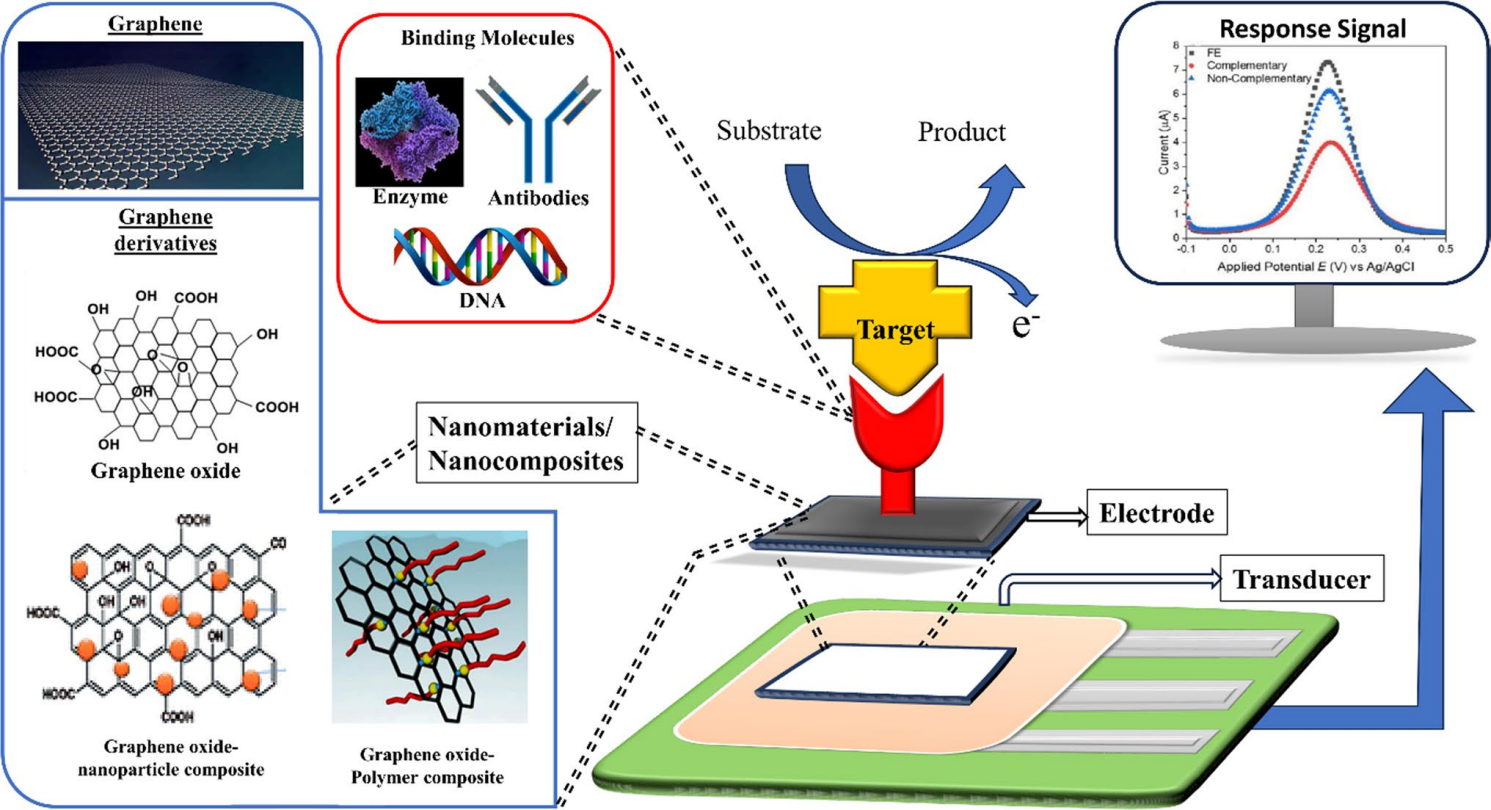
Schematic depiction of electrochemical nano biosensor: Functioning and principles for biological contaminant detection

The basic working principle of an electrochemical nano biosensor is schematically represented in Fig. 4, where the layer of biorecognition element (sensory layer) immobilized on a high surface-area nanomaterial (signal transducer) binds the biomarkers or other intended analyte and the transducer explicitly transfer this information into a determinable electrochemical signal to be detected by a processing device.

In addition, researchers like Singh et al*.*[105] have also explored different biosensor types and their applications, highlighting the advantages of integrating machine-learning techniques in this area. Negahdary et al*.*[106] demonstrated that scientists are developing electrochemical biosensors with peptides and nanomaterials to produce new and superior diagnostic procedures with higher sensitivity, selectivity, and lower production costs. The investigation proved that GCE (glassy carbon electrodes) and GE (gold electrodes) were the most popularly used signal transducers. Due to a variety of reasons, such as the availability of free amino acids, the generation of decarboxylases by microorganisms, and changes in processing and storage conditions, biogenic amines (BAs) are frequently discovered in food. Sombir Kashyap and colleagues emphasize the significance of BAs as crucial indicators for assessing food freshness and quality. They extensively discuss widely employed electrochemical transducers, specifically BA biosensors utilizing amperometric, potentiometric, impedimetric, and conductometric measurements.

**Fig. 5:**
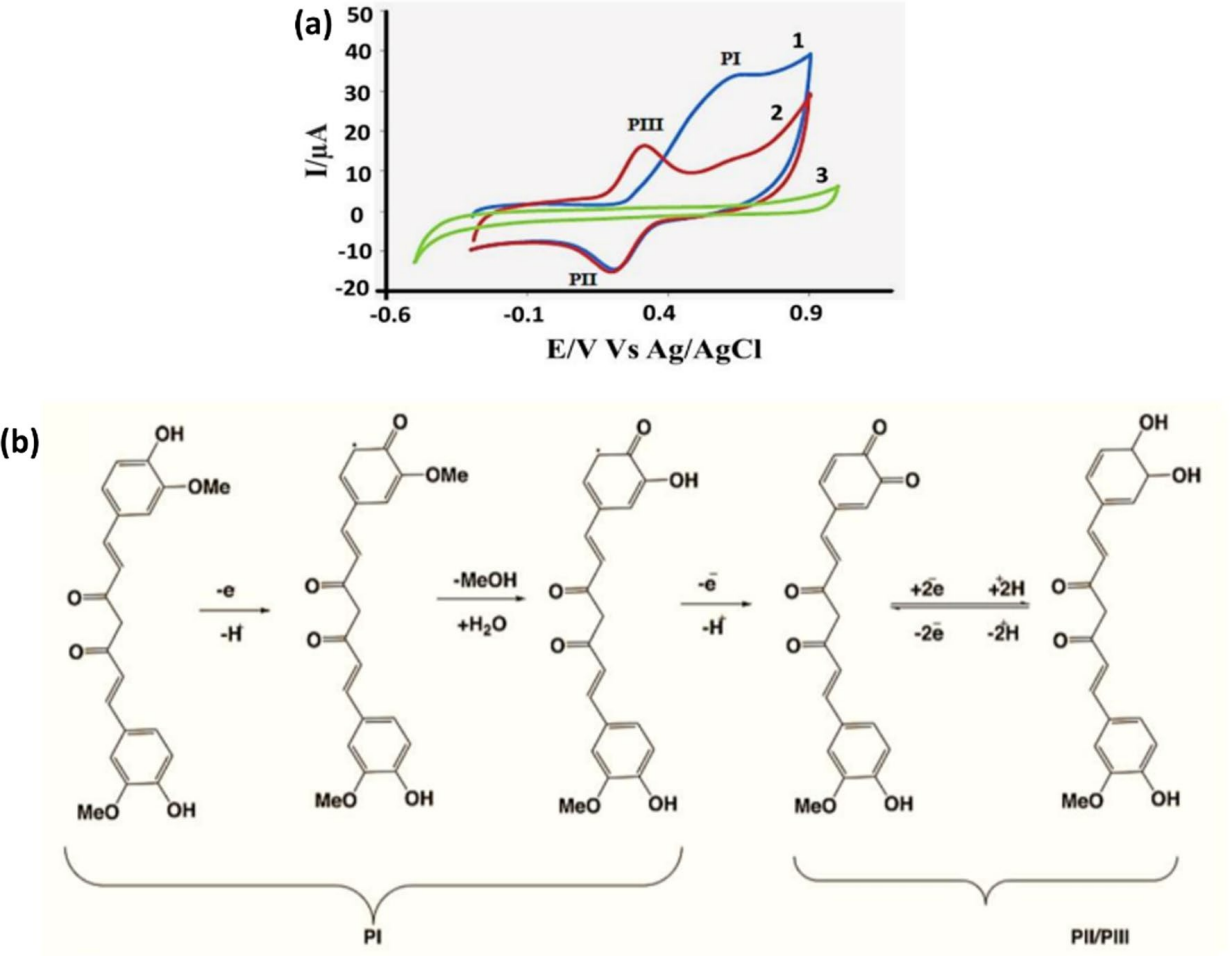
**a** Cyclic voltammograms of the GC/β-CD-rGO electrode: (1) First cycle, (2) Second cycle, both in the presence of 1 mM curcumin, and (3) in the absence of curcumin, recorded in 0.1 M phosphate buffer solution at pH 7.4 with a scan rate of 50 mV s^−1^ **b** electrochemical oxidation of curcumin [109]. Adapted from an open-access article

The researchers also incorporate noteworthy findings from previous studies [107]. Electrochemical biosensors have garnered more attention in recent years than other analytical methods including flow systems, chromatography, migration techniques, fluorescence, and spectrophotometry. These systems possess the versatility to seamlessly integrate with labs-on-chips to provide outstanding point-of-care analytical tools because they offer practicality, sensitivity, and quick response. Due to their distinctive characteristics, they are good instruments for a variety of analytes, including medicines, proteins, markers, bacteria, and viruses, among others. Bakirhan et al*.*[108] illustrated the diverse range of applications for electro-analytical biosensors. Even though disposable electrodes are now routinely employed, changes in electrodes have been the subject of more extensive research because of their increased surface area and catalytic activity. Mirzaei et al*.*[109] pioneered the utilization of a β-CD/rGO (beta-cyclodextrin–rGO) nanocomposite to establish an exceptionally sensitive electrochemical detector for the precise quantification of CM (curcumin). Figure 5a displays a cyclic voltammogram of the GC@β-CD/rGO electrode without CM and two subsequent cycles with 1 mili Molar curcumin, all set in pH 7.4. In the CM’s presence, an irreversible oxidation peak-I and a paired reduction–oxidation peak (II–III) are evident. The electrochemical oxidation of CM on the electrode surface has occurred because of the existence of phenolic -OH groups and methoxy functionalities (Fig. 5b).

Meng et al*.*[110] fabricated a flexible paper-based electrode by combining rGO and silver nanoparticles through vacuum filtration. This electrode efficiently detected Sudan II in chilli powder, demonstrating high sensitivity (22.93 A mol/L) and a low LOD (41.3 nmol/L) due to Ag NPs' electrocatalytic properties in azo bond reduction. Notably repeatable and stable, the sensor achieved 96.1%-101.8% recovery (RSD 6%). The integrated Ag NPs and rGO paper electrode present a robust platform for Sudan I detection, with heightened electroactivity and structural advantages, promising precise and sensitive analysis of food samples. The disposable electrode offers an economical and effective solution for electrochemical Sudan I detection.

Hassasi et al*.*[111] introduce a sensitive electrochemical method for evaluating anti-diabetic drug MET (metformin) in serum and pharmaceutical samples. They enhance sensitivity using a copper-graphene nanocomposite (Cu-G/CPE) on a CPE (carbon paste electrode) through multivariate optimization. Cu-G@CPE, resulting from CPE surface modification, displays features such as low background current, renewability, and cost-effectiveness. DPV, CV, and chronoamperometry investigate MET electrochemical behaviour on Cu-G@CPE, which displays superior electrocatalytic properties for MET detection and oxidation due to distinctive Cu-G nanoparticle properties. The response surface approach optimizes conditions for high oxidation current density. Cu-G@CPE excels in analytical performance, offering low LOD, vast linear range, stability, repeatability, and reproducibility for MET analysis. It also shows improved poisoning tolerance compared to unmodified CPE during MET electro-oxidation. The sensor’s cost-effectiveness, renewability, and electrocatalysis suggest pharmaceutical quality control potential. In real samples, like tablets and human plasma, Cu-G/CPE proves a promising electrochemical MET quantification sensor.

Yanik et al*.* introduced a novel biosensor using a reduced GO@palladium nanoparticles-poly (2-amino-4-chlorophenol) (rGO@Pd-PACP) nanocomposite to modify pencil graphite electrodes (PGE) for drug-DNA interaction analysis. The biosensor exhibited enhanced sensitivity, detecting interactions between anticancer drugs (Mitomycin C and Acyclovir) and double-stranded DNA. The nanocomposite-mediated electrode demonstrated a 17-fold rise in guanine oxidation signal sensitivity in comparison to unaltered PGE. The sensor achieved a low detection limit (0.0513 M or 51.3 nM) for Acyclovir, with a linear scale from 0.1 to 0.5 M. The biosensor effectively distinguished drug-DNA interactions and provided specificity for drug classification. The modified electrode displayed improved electroactive surface area and reduced surface resistance. Importantly, the biosensor exhibited high electrochemical performance, selective detection of drug-DNA interactions, and applicability for analyzing pharmaceutical preparations. The nanocomposite-based disposable biosensor offers advantages such as cost-effectiveness and portability. The study's findings highlight the biosensor's potential for sensitive DNA analysis, drug interaction determination, and future applications in anticancer drug development and mechanistic studies of drug-DNA interactions. The biosensor design represents a significant advancement in effectively discerning DNA interactions of various drugs [112].

## Graphene (GR) based nano biosensors

During the past decade, diverse nanomaterials have been harnessed to develop remarkably efficient biosensors capable of detecting analyte biomolecules. Graphene, in particular, has significantly propelled the advancement of cost-effective electrode materials in research due to its exceptional physical attributes, like excellent electrical conductivity, large specific surface area, optical transparency, high carrier mobility and flexibility.

### Graphene (GR)-based nanocomposites as biosensing material

The remarkable physicochemical properties, exceptional catalytic activity, and economical manufacturing costs of graphene nanocomposites make them highly desirable for developing advanced chemical and biological sensors for the next generation [113–116].

GR and its oxidized derivatives, including GO, encompassing diverse oxygen moieties such as epoxy, carboxyl, and hydroxyl functionalities, have appeared as promising candidates for biosensor application [18]. These functionalities display strong hydrophilic properties of GO sheets and facilitate the incorporation of diverse inorganic nanoparticles, such as metal oxides, noble metals, nanoclusters (NCs), quantum dots (QDs), and semiconducting nanoparticles. This integration serves to improve the proficiency of sensors based on GO [117, 118]. Furthermore, reducing GO into rGO introduces a substantial density of defects, resulting in significantly heightened electrochemical activity compared to Chemical vapour deposition-grown graphene. This elevated electrochemical activity proves particularly beneficial for the advancement of electrochemical biosensors. Moreover, GR-based nanocomposites possess distinctive structural configurations and features that offer valuable detection advantages [119]. The 3-D interlinked hierarchical arrangements of GR nanocomposites offer enhanced diffusion capabilities for diverse biomolecules while preserving their biocatalytic functions, thereby optimizing the functionality of biosensing [120, 121]. In the pursuit of biosensing applications, graphene-based hybrids with polymers [122, 123], and metal nanoparticles decorated on surface [117] have been extensively explored because of their outstanding generous surface area, biocompatibility, and the capacity to selectively attach with biomolecules. Various nanostructures have been investigated for biosensors, encompassing the detection of heavy metals [124, 125], glucose and hydrogen peroxide [125], nucleic acids [126], pathogenic bacteria [127], antibodies [128], cancer biomarkers [129–131] and numerous other targets [29, 132, 133]. Various electrochemical and fluorescent biosensors rely on graphene nanocomposites for their detection capabilities. These nanocomposites include GO, GR, rGO@polymer combinations, GR@inorganic NP hybrids, 3-D GR coordinated with different polymer hydrogel networks, and metal/metal oxide NPs. These diverse nanocomposites are proficient in detecting an array of analytes, like H_2_O_2_, ascorbic acid (AA), cholesterol, nucleic acids (NAs), dopamine (DA), uric acid (UA) and glucose cofactors like adenosine triphosphate (ATP) and nicotinamide adenine dinucleotide (NADH), pathogens, metal ions, pesticides, food toxins and cancer biomarkers [127–131].

By introducing heteroatoms such as phosphorus (P), boron (B), nitrogen (N), and sulfur (S) we can effectively modify the surface traits and physicochemical properties of graphene [134]. Numerous strategies, like in situ doping that synchronizes the creation and doping of GR, have been employed. This can be achieved through bottom-up synthetic approach, ball milling, or Chemical Vapor Deposition (CVD) methods. Graphene oxide (GO), showcases distinct properties in electronics, electrochemistry, thermodynamics, and mechanics. Its hydrophilic characteristics transform it into flexible, transparent, and biocompatible nanosheets. Nanosheets of GO have different functionalities that enable them to interact with many biomolecules, making them incredibly beneficial in the design of biosensors [135–137].

The fusion of GR-based materials, such as GO and rGO, with components like organic polymers, metal nanoparticles, and bioactive enzymes or proteins has arisen as a highly effective strategy. for creating advanced biosensors [122, 138, 139]. These hybrid materials harness the unique properties of their constituents, thereby expanding their potential applications. GO and rGO, in particular, offer several benefits when used to develop biosensors. Their inherent surface defects and abundant reactive oxygen group moieties are favourable sites for initiating and controlling the formation and growth of various materials, like metal oxides, metals and semiconductor nanoparticles. For example, Liu et al*.* [140] constructed bioconjugate molecules using different nanoparticles, including platinum nanoparticles (PtNPs), gold nanoparticles, palladium nanoparticles (PdNPs), silver nanoparticles (AgNPs), and latex. They achieved this by leveraging the BSA (bovine serum albumin)-mediated reduction and attachment of these nanoparticles in nanosheets of GO. The concentration of the AuNPs present in the resulting BSA@GO composite could be administered by simply varying the quantity of BSA protein used.

### Graphene-based fluorescent biosensors

Fluorescence-dependent biosensors offer numerous benefits, such as elevated sensitivity, rapid response times, and simplicity of use, making them incredibly valuable in biological and medical research fields. GR-based materials like graphene oxide (GO) have become particularly notable in this context due to their potent fluorescence quenching abilities [141]. The quenching of fluorescence is primarily associated with the FRET (Forster Resonance Energy Transfer) mechanism, which is influenced by the unique linear distribution of Dirac e^−^s in graphene. This, in combination with a sufficiently large separation between the fluorescent molecules and the graphene surface, allows for effective energy transfer [142]. Graphene's capability to proficiently quench the fluorescence of a broad spectrum of fluorophores makes it an attractive material for the enrichment of innovative fluorescent-dependent biological sensors. These sensors can be employed in various in vivo and in vitro applications [143–145]. An efficient energy transfer occurs when biomolecules are adsorbed onto a GO nanosheet and conjugated with a fluorescent dye. This signifies the quenching of the fluorescent signal, giving rise to a minimal background signal. The lack of an energy bandgap in graphene and nearby π-π stacking between the graphene and adsorbed biomolecules are key to this phenomenon.

#### Detection of pathogens and food toxins using graphene-based biosensors

Due to their excellent fluorescence quenching capabilities, GR-based nanomaterials have become a focus for designing aptamer-induced fluorescence quenching sensors. These sensors are particularly beneficial for sensing toxins and pathogens found in food [146].

Lu and his colleagues developed a notable example of such a sensor [147]. They created an aptamer-based fluorescence recovery test to detect AFB1 (aflatoxin B1), a harmful mycelial toxin produced by the Aspergillus flavus fungus. Their system used a FRET-based quenching mechanism involving GO and CdTe QDs. In their setup, an AFB1-specific aptamer with a thiol group was attached to the CdTe QDs, resulting in quenched fluorescence due to GO. However, upon the introduction of AFB1, the fluorescence was renewed proportionately to the amount of AFB1 present. The constructed CdTeQDs/GO sensor demonstrated a broad detection range between 3.2 nM and 320 μM and a low detection limit that is 1.0 nM., proving its effectiveness even in complex mediums like peanut oil solution.

Another exemplary development was by Shi and the team [148], who contracted a FRET-based biosensor for sensing the bacterium *Staphylococcus aureus*. Their sensor involved using GQDs and AuNPs, achieving an impressive fluorescence quenching efficiency of 87%. This biosensor was designed to anchor the seize probes on the GQDs while reporter probes were attached to the Au nanoparticles. The subsequent formation of a sandwich-like structure through co-hybridization of the target oligomers with the capture probes initiated the FRET mechanism, allowing for precise pathogen detection.

#### Detection of glucose, cholesterol, hydrogen peroxide, and dopamine

Applications of nanocomposites containing GR, for fluorescence-based detection are proliferating, particularly in the realm of substances such as H_2_O_2_, C_6_H_12_O_6_ and dopamine (DA), as they showcase superior sensitivity and specificity. Chen and his team [149] utilized a graphene oxide (GO)-based label-free sensor operating in the near-infrared (NIR) spectrum for fluorescent identification of dopamine (DA). When DA molecules attach to the GO nanosheet, the efficient quenching of GO allows for biosensing via the direct observation of NIR fluorescence emanating from the GO, leading to efficient DA detection. The sensor displayed a lower LOD at 94 nM and a relative standard deviation of 2.0%, demonstrating its practicality for determining DA in biological samples with an associated recovery rate between 98 to 115%. Xiaofei [150], and his associates have synthesized gold nanoclusters@glucoseoxidase-GO (GNC/GOD/GO) versatile catalyst and utilized it in the fluorescent detection of glucose. The fluorescence peak intensity exhibits a consistent linear decrease as the C_6_H_12_O_6_ concentration increases within the scale of 1.1 × 10^–2^ to 1.6 × 10^–7^ M with the limit of detection 6.8 × 10^–8^ M. Another research group, led by Li [151], created a nanocomposite of tyramine (TYR)-functionalized graphene quantum dots (GQDs), denoted as TYR-GQDs, aimed at fluorescent biosensing of H_2_O_2_ and other vital metabolites: L-lactate, C_6_H_12_O_6,_ xanthine and cholesterol.

**Fig. 6:**
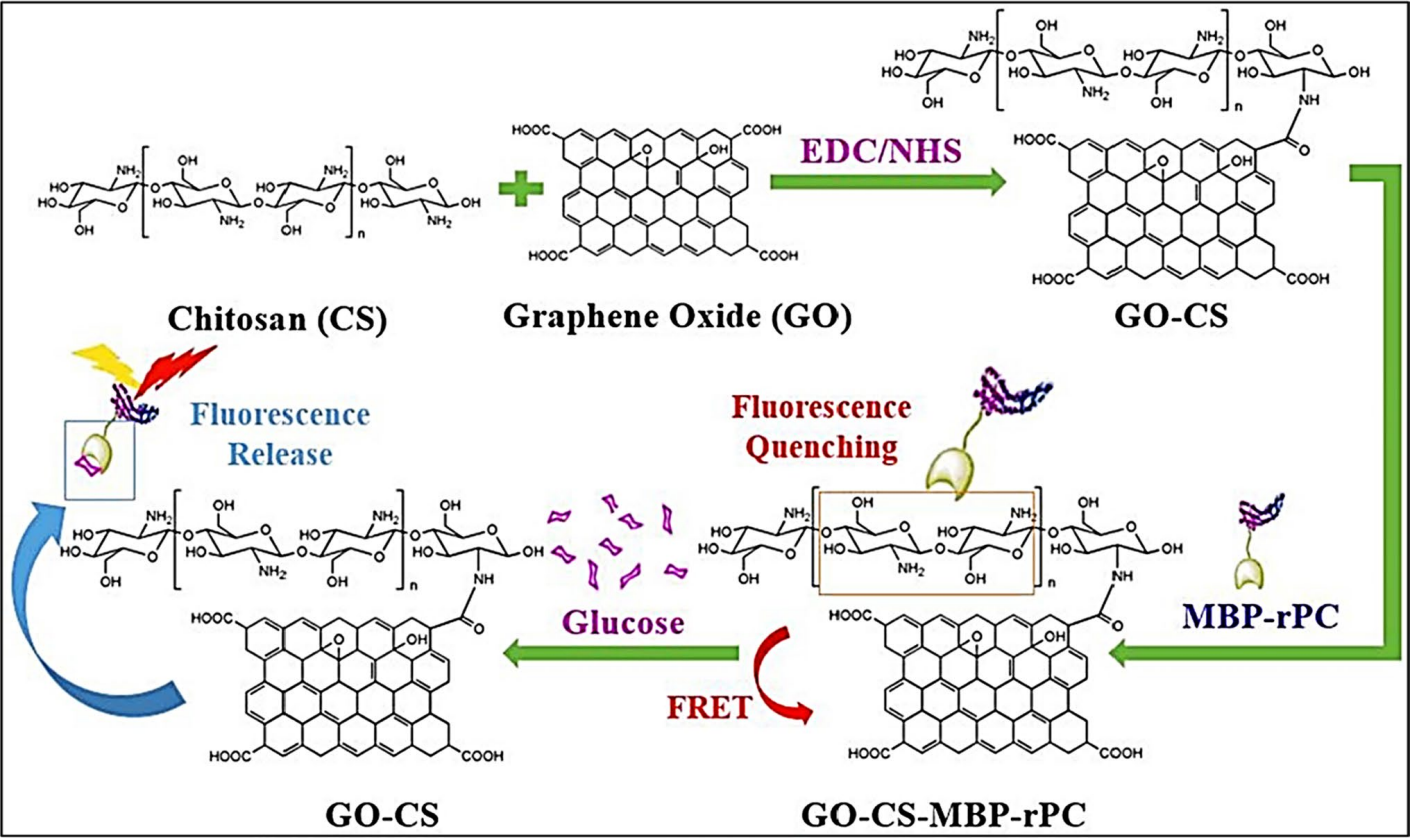
Detection of C_6_H_12_O_6_ using a biosensor based on MBP-rPC and GO-CS. Adapted from open access article [152]

In this composite, TYR is covalently bonded to the GQDs surface by reacting with TYR's amino groups (–NH_2_) and GQDs' carboxyl groups (–COOH). For sensing of these metabolites, specific oxidases, including L-lactate oxidase, xanthine oxidase, cholesterol oxidase, and glucose oxidase, were introduced into a 10 mM phosphate-buffered saline of pH 7.0, incorporating 0.1 mg ml^−1^ TYR-GQDs conjugate. This procedure triggered a photoluminescence quenching response, enabling the successful detection of the four metabolites. Figure 6 represented the biosensing glucose using a system based on MBP-rPC and GO-CS (MBP- maltose-binding protein, rPC- α-subunit of recombinant Phycocyanin and CS-Chitosan). In this case, GO-CS composite adsorbed MBP-rPC non-specifically which resulted in the fluorescence quenching of GO-CS. Where, after the addition of glucose, the quenching effect of GO-CS on MBP-rPC was suppressed because glucose out-compete the interaction of GO-CS with MBP-rPC which leads to a significant increase in fluorescence. The biosensor showed efficient sensitivity for glucose sensing with a linear range of 0.1–1 mg/ml along with the LOD, 0.05 mg/ml [152].

### Graphene (GR)-based electrochemical biosensors

GR-based materials in electrochemical sensors provide a swift electron transfer, particularly at structural edges and irregularities, compared to the basal planes. These inherent structural irregularities in chemically altered graphene can be utilized as electrochemical sensors [153, 154]. The exceptional surface area and electrical conductivity of GR establish it as a suitable candidate for electrode material. It also boasts impressive electrochemical attributes, such as expansive potential windows, rapid electron transfer, resistance, high electrochemical activity, and minimal charge-transfer [155, 156].

Graphene's unique characteristics, such as two-dimensionality, robustness, compact size, and the ability to combine it with different carbon-based materials, ensure greater integration possibilities. With superior electrochemical and physical properties, GR is a prime component for electrochemical detection applications. The graphene family (GO, rGO, and GQDs) is extensively utilized in creating biosensors, energy storage devices, and drug delivery systems because of their exceptional conductivity, biocompatibility, and superior mechanical performance [157–160]. Furthermore, oxygen-containing moieties facilitate potent functionalization with numerous polymers and biomolecules for diverse applications [161, 162]. In addition, these nanomaterials offer a vast surface area, which enhances the sensitivity of the biosensor by amplifying the immobilization of receptors [163, 164].

#### Detection of pathogens

Water pollutants primarily consist of harmful pathogens like viruses, bacteria, protozoan parasites, and fungi, reliable for different waterborne diseases. The existence of these microorganisms in both water and food sources presents a significant health risk because of their rapid proliferation and detrimental impact on human health [165–167]. Moreover, the infectious agents, which include viruses, bacteria, fungi, viroids, nematodes, and phytoplasmas result in infectious diseases which cause a worldwide reduction in agricultural yield [168]. Enterohemorrhagic strains of the bacterium Escherichia coli are the most severe among the other pathogens, causing serious foodborne outbreaks [169]. In response to these threats, nanomaterials like GR and CNTs are used to construct electrochemical biosensors. These devices enable selective and sensitive sensing of harmful pathogens [127]. Pandey and his group of researchers [170] have presented a graphene-centric biosensor, constructed without labels, that is precisely tailored for identifying pathogenic bacteria. This detection device employed graphene in conjunction with micro-scaled interdigitated electrodes and was bio-enhanced with antibodies explicitly targeting the *E. coli* O157-H7 strain. Fluctuations in the device’s capacitance demonstrated its sensitivity, facilitating the sensing of the *E. coli* O157-H7 strain in concentrations ranging from 10 to 100 cells per millilitre.

**Fig. 7:**
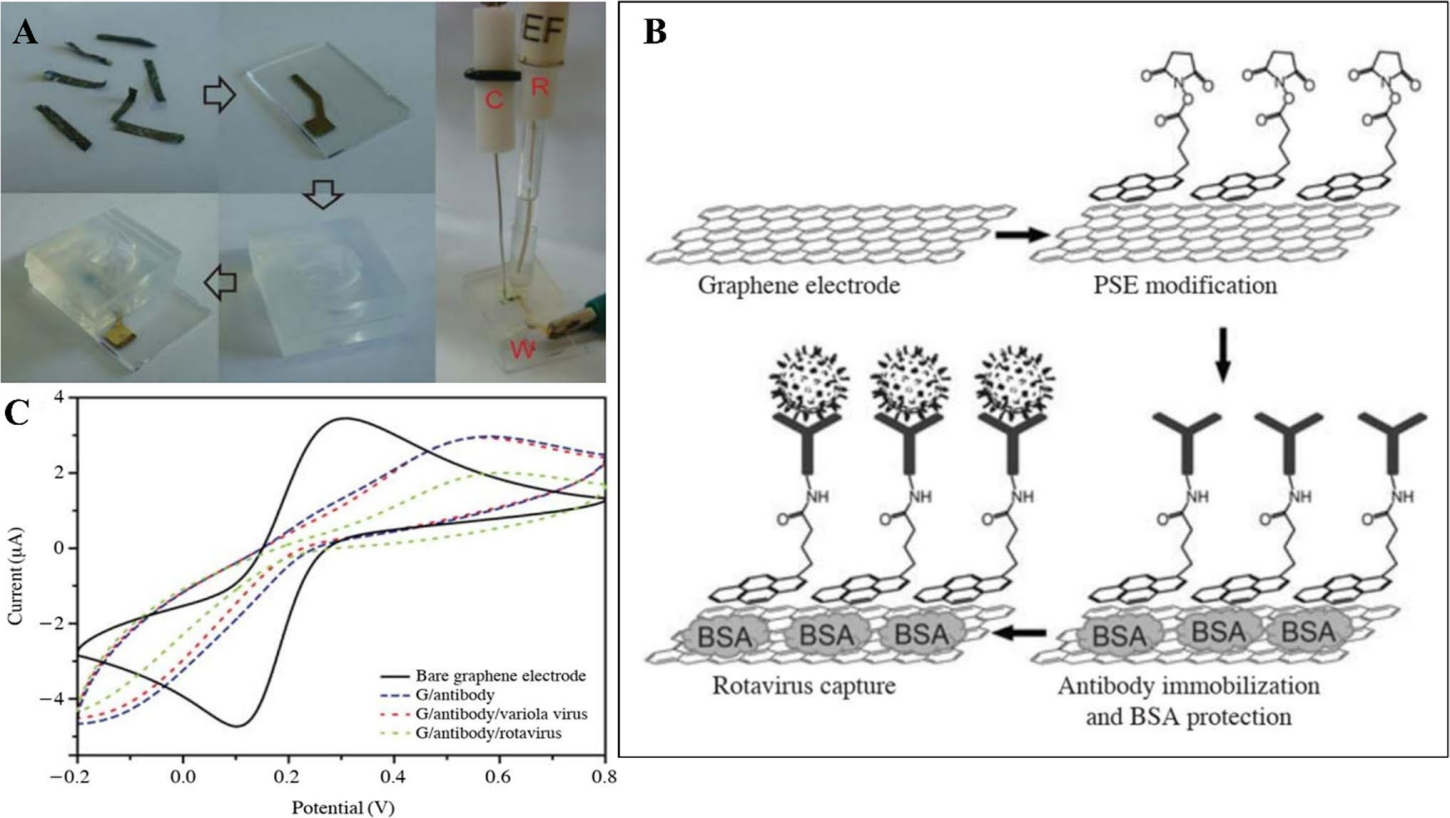
**A** The three-electrode cyclic voltammetry (CV) system was constructed utilizing a GR film as the working electrode **B** Demonstration of the graphene film-induced immuno-biosensor designed for the sensing of rotavirus. **C** Cyclic voltammograms were obtained for various electrodes: bare graphene electrode, G@antibody/modified electrode, G@antibody/rotavirus with 10^5^ pfu/mL rotavirus incubation, G@antibody-variola virus used as a negative control procedure [171]

Liu et al.[171] fabricated a highly responsive biosensor using graphene for virus detection. They created a conductive GR film through a two-step process involving graphene oxide (GO) film formation and thermal annealing (Fig. 7). This graphene film exhibited excellent electron transport properties, serving as a working electrode for an electrochemical biosensor. Pyrene derivatives were used for surface modification, allowing covalent attachment of virus-specific antibodies. The biosensor successfully detected rotavirus with a sensitivity of 30.7% at 10^5^ pfu/mL and 1.3% at 10^3^ pfu/mL. The investigation highlights the potential of GR-based immunobiosensors for utilisation in clinical diagnostics, environmental monitoring food safety.

#### Detection of cholesterol

High cholesterol levels in the blood are recognized as a severe risk factor for cardiovascular illnesses, including heart attacks, atherosclerosis, heart disease, and hypertension [172]. A detailed analysis has been performed on using GR-based nanocomposites to develop enzyme-oriented cholesterol detectors. For instance, Dey et al*.* [173] developed a biosensor that used redox enzyme-modified GO, which they accomplished by covalently linking ferrocene redox entities to the graphene oxide framework using diamine, leading to an improved e-transfer rate. The response rate of this biosensor was recorded as 5.71 μA μM^−1^ cm^−2^, and it displayed a linear shift from 0.5 to 46.5 μM, with a lower sensing limit of 0.1 μM.

**Fig. 8:**
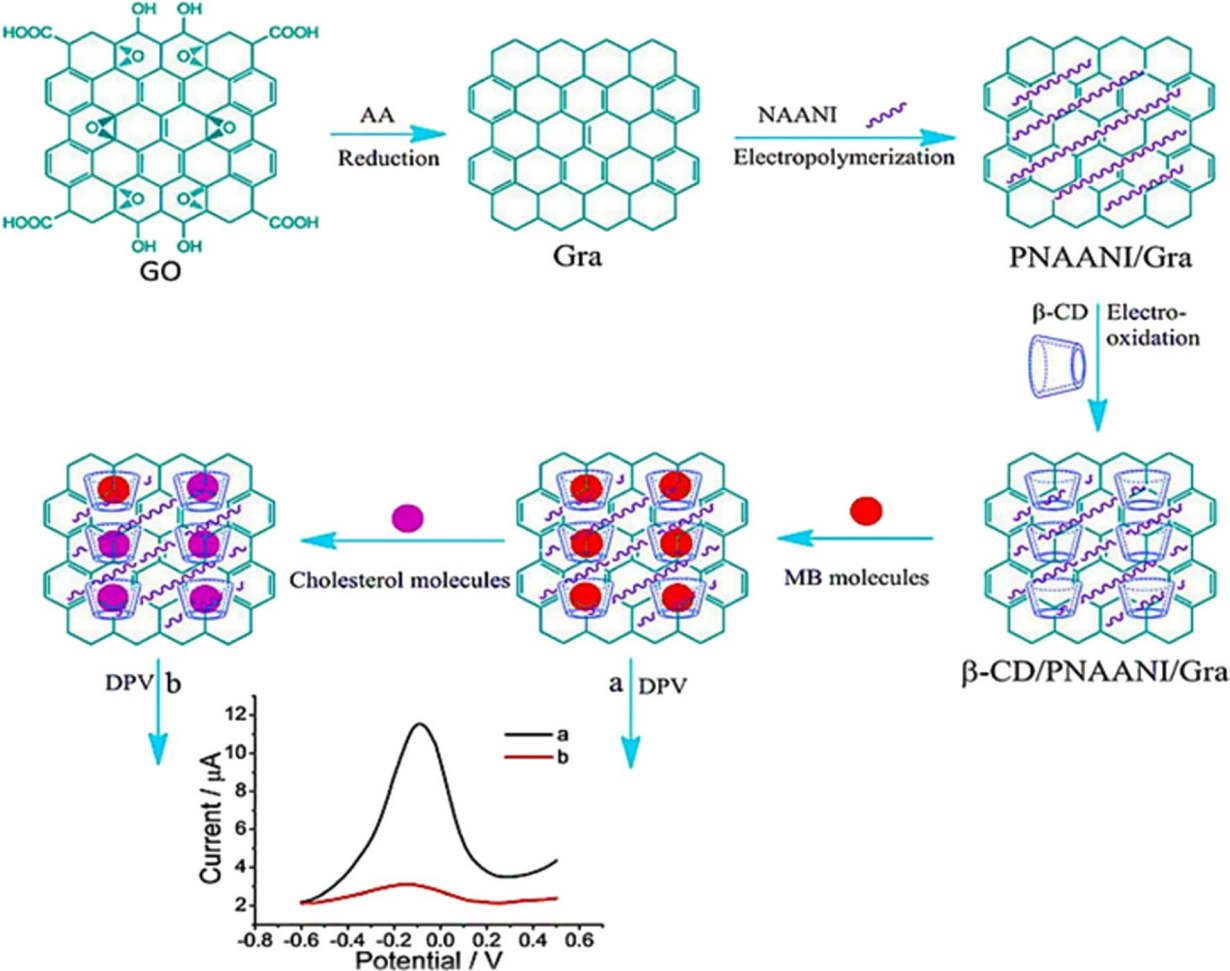
Depiction of the approach underlying the suggested electrochemical sensor, characterized by a competitive host–guest interface involving (β-CD) β-cyclodextrin, methyl blue (MB) acting as the signal probe, and cholesterol as the target molecule. [174] Adapted from the article

Yang et al*.* have devised an innovative electrochemical technique for highly specific and sensitive cholesterol detection. The method capitalizes on competitive host–guest interactions involving β-cyclodextrin, which interacts with either the aimed molecule (cholesterol) or a signal probe (methylene blue, MB). This strategy employs a customized electrode, denoted as β-CD@poly(*N*-acetylcholine)-GR (β-CD/PNAANI-GR), which utilizes the hydrophobic interior of β-CD to accommodate MB. As a result, an augmented anodic peak is observed on the modified electrode. When cholesterol is present, its competitive interaction displaces MB, leading to the decrease of the oxidation peak. This modification enables the precise detection of cholesterol using differential pulse voltammetry, with an impressive low LODs of 0.50 mM and a linear detection scale spanning 1.00–50.00 mM. The approach exhibits exceptional analytical prowess across a wide linear range and displays significant selectivity against potential interferences. Notably, this pioneering method eliminates the requirement for enzymes or antibodies, offering a promising avenue for effective cholesterol detection. Moreover, it provides valuable insights into the intricate interactions between cholesterol and β-CD (Fig. 8) [174]. Li and his associates [175] demonstrated a cholesterol biosensor that utilized a chitosan biopolymer@graphene (CS@GR) nanocomposite. The presence of –OH and –NH_3_/NH_2_ side groups in the chitosan biopolymer supported stable conjugation with the cholesterol oxidase enzyme (ChOx). The biosensor based on ChOx/CS-GR demonstrated a linear sensing scaled from 0.005 to 1.0 mM and a detection limit at 0.715 μM specifically for cholesterol analysis. Additionally, it displayed a distinct selectivity against potential interfering bio-analytes, which include uric acid (UA), dopamine (DA), glucose, and ascorbic acid (AA). Cholesterol biosensors have been reported that utilize hybrid nanostructures of Pt nanoparticles with graphene (PtNPs/GR) [176] and Pd nanoparticles with rGO (PdNPs/rGO) [176] for electrochemical cholesterol detection. The biosensor employing the PdNPs/rGO hybrid nanostructure exhibited a heightened response rate of 5.12 μA μM^−1^ cm^−2^ and a detection limit of 0.05 μM, due to the superior electrocatalytic properties of the dendritic Pd nanoparticles on the surface of rGO.

#### Detection of glucose

There has been a notable increase in enzyme-based electrochemical glucose sensors throughout the preceding forty years due to their notable precision, sensitivity, and simplicity 16. The employment of GR-based materials in these sensors is particularly noteworthy attributable to their biocompatibility, significant surface area, exceptional electrochemical properties, and abundant oxygen-bearing functional groups, including epoxy, carboxyl, hydroxyl, and carbonyl groups. These properties contribute to the successful fixation of redox enzymes, facilitating glucose detection through either physical adsorption or covalent linkage [114]. To boost the catalytic performance of the anchored glucose oxidase (GOx) and the robustness of the bio-sensor, GO is amalgamated with a range of polymers, like PVP (polyvinylpyrrolidone) [177], biopolymers like CS (chitosan) [178], and PEG (polyethene glycol) [179]. Liu and his associates unveiled a straightforward, singular-step method for developing enzymatic glucose biosensors anchored in chitosan-based electrodeposition [180]. The development of the biosensor involved the electrodeposition of a compound consisting of an Fc-CS (ferrocene-enriched chitosan hybrid) and a solution of SWCNT (single-walled carbon nanotubes) to yield a uniform film populated using glucose oxidase enzymes. This blend of Fc-CS@SWCNT@GOx was anchored on the 3D graphene foam (GF) surface using electrodeposition. Owing to the outstanding features of the 3D GF, which encompasses high electrical conductivity, rapid mass transport dynamics, and a vast active surface area, the sensor displayed an extensive linear response across varying concentrations spanning from 5.0 μM to 19.8 μM. It also indicated a LOD of 1.2 μM and expedited feedback, achieving 95% of its total response in less than 8 s, proving its efficiency in glucose detection.

The combination of graphene and inorganic nanoparticles garnered considerable attention and is integral to creating composite nano architectures employed in electrochemical glucose sensing [18, 117, 181]. Claussen et al*.* [182] illustrated a biosensor that had platinum nanoparticles (PtNPs) adorned on the MGPNs (multilayered graphene petal nanosheets). By modifying both the structure and size of electrodeposited PtNPs, they optimized the biosensor's performance. This specially constructed GOx-PEDOT@PtNP@MGPN bio-sensor showcased a substantial linear scope for detecting glucose from 0.01 to 50 mM with a detection threshold of a minimal 0.3 μM. It also displayed longevity by preserving 75% of its sensitivity for 5 weeks. Çakıro˘glu and Ozacar [183], in a different study, constructed a glucose bio-sensor utilizing TA (tannic acid) coating with AuNPs onto rGO nanocomposites. The constructed biosensor demonstrated a sensitivity of 18.73 mA mM^−1^cm^−2^ and demonstrated a linear detection domain from 2 to 10 mM.

#### Detection of cancer biomarkers

A biomarker refers to a biological aspect denoting standard or anomalous biological activities, disease processes, or physiological reactions to introduced treatments [184]. Nanomaterials present promising opportunities as mediums for cancer diagnosis and treatment. Consequently, research has transitioned from initial, preclinical phases to practical point-of-care implementations [185]. Graphene and graphene oxide (GO) are highly desirable materials in biomarker detection because of the simplicity of their surface modification chemistry and their distinctive chemical, electrical, optical, and electrocatalytic attributes [186, 187].

In addition, metal nanoparticles, like AuNPs, have been implemented in developing electrochemical biosensors as immobilization platforms, leveraging their high electrical conductivity, superior adsorption capabilities, high stability, and simplicity of surface modification [188]. Zhu and his team [189] engineered an electrochemiluminescence immunosensor that utilizes a CdSeQD-adorned poly(diallyl dimethylammonium chloride)/GR@AuNPs hybrid composite for detecting ractopamine. The sensing material exhibited a linear detection scope from 0.01 to 1000 ng mL^−1^ also a lower sensing limit of 2.6 picograms mL^−1^. It demonstrated considerable stability and sensitivity in the detection of biomarkers. Feng and his associates [190] developed a sandwich-style immunosensor to sense PSA (Prostate-Specific Antigen) a marker for cancer. This device utilized gold nanoparticles (AuNPs) anchored on thionine-mediated GO. Furthermore, bimetallic platinum copper NPs were incorporated on rGO and g-C_3_N_4_, serving as markers for secondary antibody binding and signal intensification. This specially formulated immunosensor demonstrated a linear sensing capability from 50 fg mL^−1^ to 40 ng mL^−1^, with an LOD of 16.6 fg mL^−1^ for PSA biomarker detection. In Table 1, we have summarized some GR-based fluorescent and electrochemical biosensors for identifying metal ions, pathogens, pesticides, food toxins, glucose, cholesterol, and cancer biomarkers, along with their linear sensing range and LODs. Modified graphene-based nanocomposites showed superior sensing attributes to pristine graphene and its derivatives. Among several modified GR-based nano biosensors noted in the table, metal-polymer modified graphene-based biosensor, i.e., Graphene/Polypyrrole/ AuNPs, shows excellent performance in sensing breast cancer biomarker, miRNA-21, with a uniform sensing capacity spanning from 1.0 fM to 1.0 nM. The sensor demonstrated a lower LOD of 0.02 fM [191].

**Table 1:** Graphene-based fluorescent and electrochemical biosensor

Graphene-based nanocomposite	Biosensor type	Target	Linear range	Limit of detection	References
GQDs/AuNPs	Fluorescent biosensor for Pathogen	*S. aureus*	100 pM to 100 nM	1 nM	[148]
GQDs-AuNPs/Indium tin oxide	Electrochemical biosensor for food toxin	AFB1	0.1–3.0 ng mL^−1^	0.008 ng mL^−1^	[192]
GLY-GQDs- Ce^4+^	Fluorescent biosensor for biomolecule	Ascorbic acid	0.03–17.0 µM	25 nM	[193]
rGO/Fe_3_O_4_ NPs	Enzymatic electrochemical biosensors	Glucose	0.5–12 mM	0.05 mM	[194]
rGO-CNT	Electrochemical biosensor for pathogen detection	*S. Typhimurium*	10^1^ until 10^8^ cfu mL^−1^	10^1^ cfu mL^−1^	[195]
Tyramine-functionalized GQDs	Fluorescent biosensors for detecting biomolecules	Cholesterol	80 nM to 10 µM	1.2 nM	[151]
N-doped GQDs MIP	Fluorescent biosensors for detecting Pesticides	Thiacloprid	0.1–10 mg L^−1^	0.03 mg L^−1^	[196]
rGO/dendritic Pd nanostructure	Electrochemical biosensors	Cholesterol	0.005–0.014 mM	0.05 µM	[197]
Polyaniline/GO fibrous nanocomposite	Electrochemical biosensors	Dopamine	2–18 µM	0.5 µM	[198]
PtPd/N-doped GR-QDs/AuNPs	Fluorescent biosensors for detecting different cancer biomarkers	CEA (Carcinoembryonic antigen)	5 fg mL^−1^ to 50 ng mL^−1^	2.0 fg mL^−1^	[199]
rGO-Cu-CuO-Cu_2_O	Electrochemical biosensor for detection of cancer biomarker	Pyruvic acid	5–370 µM	1.21 µM	[200]
AuPt/GQDs NCPs	Fluorescent biosensors for biomolecule detection	Glucose	10^−10^–1 M	10^−10^ M	[201]
PDI-HIS–Cu–GO nanocomposites	Fluorescence biosensor for cancer biomarker detection	Pyrophosphate (PPi)	0–0.33 μM	0.60 × 10^–7^ M	[202]
nZrO2@RGO	Electrochemical biosensor for food toxin detection	Aflatoxin B1, AFB1	1.5–18 ng mL^−1^	2.54 ng mL^−1^	[203]
GO-Ionic liquid-AuNPs	Electrochemical immunosensor for cancer biomarker detection	CD_44_ antigen	5.0 fg mL^–1^ to 50.0 μg mL^–1^	1.90 fg mL^–1^	[204]
poly(3-aminobenzylamine)/MoSe_2_/ /GO	Electrochemical biosensor for cancer antigen	microRNA-2	0–1000 pM	1.2 fM	[205]
N-GQDs-aptamer	Fluorescent biosensor	Tetracycline	1–100 ng mL^−1^	0.95 ng mL^−1^	[206]
CGO-Au-PtBNPs	Electrochemical biosensor	MUC1 protein	1 fM–100 nM	0.79 fM	[207]
NGQDs	Electrochemical biosensor for cancer biomarker detection	MCF-7 cells	20–106 cells mL^−1^	2 cells mL^−1^	[208]
LDH-GQDs	Fluorescent biosensor for biomolecule detection	Ascorbic acid	5–300 μmolL^−1^	1.72 μmolL^−1^	[209]
3D-rGO/Au NPs	Electrochemical biosensor	Ochratoxin A	1 pg/mL to 10 ng/mL	0.34 pg/mL	[210]
BiPO_4_@GO-MMIPs	Fluorescent biosensor for detection of antibiotic	Ciprofloxacin	39.0–740.0 μg L^−1^	0.40 μg L–1	[211]
F, N co-doped GQDs	Fluorescent biosensor	Doxycycline	0.04–100 μM	40 nM	[212]
Graphene/PPY/ AuNPs	Electrochemical biosensor for cancer antigen detection	miRNA-21	1.0 fM to 1.0 nM	0.020 fM	[191]
GQD/AuNC	Fluorescent biosensor for biomolecule detection	Glucose	1–15 μM	0.15 μM	[^213^]
Ag/TiO_2_/rGO	Electrochemical immunosensor for detection of cancer antigen	CA 15–3	0.1–300 μmL^−1^	0.07 μmL^−1^	[214]
S-GQD	Fluorescent biosensor	Omethoate	0.001 ppm–200 ppm	1 ppb	[215]
GQDs/C-dots	Fluorescent biosensor	Imidacloprid	5–4000 × 10^−9^ mol L^−1^	8.23 × 10^−10^ mol L^−1^	[216]
GQDs/AuNPs	Fluorescent biosensor	Gentamicin	1.03–16.55 μM	0.493 μM	[217]
GQDs/ Pd NPs	Fluorescent biosensor	Tetracycline	40–90 ng mL^−1^	45 ng mL^−1^	[218]

## Challenges of graphene-based bio-functional interfaces and their properties

As already mentioned in the above sections how graphene, a single layer of carbon has garnered significant attention in the field of biosensors, and the immense potential of integrating graphene into bio-functional interfaces for biomedical applications. But to harness the full potential of graphene-based biosensors we must address various challenges related to its biocompatibility, surface modification, and nonspecific interactions and ways by which the scientific community tried to mitigate the following challenges to give the readers a basic jest of it.

One of the primary concerns in the utilization of graphene in bio-functional interfaces poses a multifaceted challenge primarily rooted in its intricate biocompatibility considerations. Pristine graphene's (PG) cytotoxicity, characterized by its sharp edges and expansive surface area, necessitates meticulous evaluation. The heterogeneous toxicity profiles observed among commercially available graphene products underscore the complexity of this issue, with variations attributed to diverse factors such as size, shape, surface charge, and chemistry. Impurities and contaminants introduced during graphene production further compound biocompatibility assessment challenge [219]. Notably, distinct toxicological responses are evident between graphene oxide (GO) and pristine graphene (PG), with differential impacts on adherent and non-adherent cellular systems. Additionally, size-dependent effects on cellular uptake and internalization highlight the nuanced interplay between graphene dimensions and cytotoxicity. Surface modifications, particularly oxygen functionalization, offer a promising avenue to enhance solubility and mitigate adverse cellular interactions [220, 221]. Nevertheless, the persistence of residual contaminants underscores the ongoing complexity in aligning graphene's remarkable properties with stringent biocompatibility requirements, emphasizing the imperative for sustained research efforts to elucidate and address these intricacies comprehensively.

The modulation of graphene's hydrophilicity presents significant challenges with profound implications for its biomedical applications. Pristine graphene's inherent hydrophobicity renders it prone to aggregation and the formation of irreversible complexes in aqueous biological environments [222]. Overcoming this challenge requires strategies to enhance graphene's hydrophilicity, thereby preventing nonspecific adsorption of biomolecules and improving sensor specificity. However, achieving optimal hydrophilicity entails navigating several hurdles. Firstly, while methods such as oxygen plasma treatment, chemical functionalization with hydrophilic groups (–OH, –COOH), or polymer wrapping show promise, they often entail complex and expensive processes. Moreover, the introduction of hydrophilic functional groups may alter graphene's intrinsic properties, potentially impacting its performance in biomedical applications [223, 224]. Additionally, concerns regarding potential toxicity persist, as hydrophilic graphene materials, while offering improved biocompatibility, may still induce cytotoxic effects and elicit immune responses in biological systems [225]. Standardization of purification procedures is imperative to ensure the removal of impurities and contaminants, which can influence graphene's hydrophilicity and biocompatibility. Addressing these challenges is essential for harnessing the full potential of hydrophilic graphene in biomedical applications, where its interactions with biomolecules play a critical role in sensing, drug delivery, and tissue engineering endeavours.

The challenges posed by the nonspecific adsorption of graphene in biosensing applications are significant, primarily due to the potential for false positives or interfering signals, which can compromise sensor accuracy and reliability. Nonspecific adsorption occurs when biomolecules bind to graphene surfaces indiscriminately, leading to interference with target analyte detection [226]. To mitigate this challenge, surface passivation techniques such as blocking with biocompatible polymers like bovine serum albumin (BSA) or polyethene glycol (PEG) are employed to create a protective barrier between the graphene surface and surrounding biomolecules [227, 228]. Additionally, the design of selective biofunctionalization strategies is crucial for enhancing sensor specificity. This involves the site-specific immobilization of bioreceptors onto graphene surfaces through covalent bonding or affinity interactions, enabling precise detection of target analytes while minimizing interference from non-target species [229–231]. Despite these efforts, challenges persist, including the need for robust biosensors adaptable to various operating conditions, concerns regarding biofouling and protein adsorption, and the necessity for standardization of graphene materials used in biosensor fabrication [232, 233]. Moreover, considerations regarding the potential toxicity and biocompatibility of graphene materials further underscore the complexity of addressing nonspecific adsorption in biomedical applications. While ongoing research endeavours aim to overcome these challenges, ensuring the accuracy and reliability of graphene-based biosensors remains a critical objective for advancing their practical utility in biomedical diagnostics and therapeutics.

## Role of graphene-based materials in overcoming challenges in nanobiosensing

Graphene-based materials managed the several critical challenges commonly faced in traditional biosensing methodologies, such as.

- Interference and cross-reactivity: Graphene-based materials possess efficient selectivity and specificity that effectively reduce the interference originating from cross-reactivity and non-specific binding. This leads to the precise detection of targeted analytes even in the presence of interfering substances.
- Detection limit: the large surface area and superior sensitivity of GR-based materials enable the detection of low-abundance biomarkers with exceptional Limit of Detection (LOD). This would significantly improve the early disease diagnosis and the accurate detection of trace analytes within complex biological matrices.
- Biological compatibility: The biocompatibility of graphene-based materials reduces the adverse effects on biological systems. As a result, these materials could be easily incorporated into biomedical devices and biosensing platforms for various applications.

## Conclusion and future scope

In conclusion, graphene and its derivative nanomaterials hold remarkable potential to further widen the horizons of biosensor applications in the forthcoming times. The superior properties of graphene like large surface area, higher electrical conductivity and biocompatibility, facilitate the fabrication of ultrasensitive and selective biosensors. These multifunctional materials enable molecular diagnostics and point-of-care diagnosis for detecting various diseases and have regularly monitored food, health, and environmental hazards. Different types of graphene-based nano biosensors, such as optical, electrical, and electrochemical biosensors have been discussed in the review, emphasizing their advantages and limitations. In biomedical research, graphene-based nano biosensors have been widely used in biomarker detection, diagnosis of cancer cells, and the sensing of pathogenic microorganisms. Furthermore, the summarized table focuses on graphene and its composite materials employed in biosensing applications to provide novel insights to researchers, encouraging the development of advanced nanocomposites with enhanced detection capabilities. The current status of nano biosensors leaves immense scope for further improvement in clinical applications and sensitivity. Therefore, one can aptly conclude that integrating nanotechnology with biotechnology to boost sensor technology will emerge as a demanding and hot area of research in the years to come.
